# Amelioration of Glucose Control Mobilizes Circulating Pericyte Progenitor Cells in Type 2 Diabetic Patients with Microangiopathy

**DOI:** 10.1155/2012/274363

**Published:** 2012-02-28

**Authors:** Gian Paolo Fadini, Patrizia Mancuso, Francesco Bertolini, Saula de Kreutzenberg, Angelo Avogaro

**Affiliations:** ^1^Department of Clinical and Experimental Medicine, University of Padova, 35128 Padova, Italy; ^2^Venetian Institute of Molecular Medicine, 35129 Padova, Italy; ^3^European Institute of Oncology, 20141 Milan, Italy

## Abstract

Chronic diabetic complications result from an imbalance between vascular damage and regeneration. Several circulating lineage-committed progenitor cells have been implicated, but no data are available on pericyte progenitor cells (PPCs). Based on the evidence that PPCs increase in cancer patients after chemotherapy, we explored whether circulating PPC levels are affected by glucose control in type 2 diabetic patients, in relation to the presence of chronic complications. We enumerated peripheral blood PPCs as Syto16+CD45−CD31−CD140b+ events by flow cytometry at baseline and after 3 and 6 months of glucose control by means of add-on basal insulin therapy on top of oral agents in 38 poorly controlled type 2 diabetic patients. We found that, in patients with microangiopathy (*n* = 23), the level of circulating PPCs increased about 2 fold after 3 months and then returned to baseline at 6 months. In patients without microangiopathy (control group, *n* = 15), PPCs remained fairly stable during the whole study period. No relationship was found between change in PPCs and macroangiopathy (either peripheral, coronary, or cerebrovascular). We conclude that glucose control transiently mobilizes PPCs diabetic patients with microangiopathy. Increase in PPCs may represent a vasoregenerative event or may be a consequence of ameliorated glucose control on microvascular lesions.

## 1. Introduction

Chronic diabetic complications are thought to result from the detrimental effects exerted by hyperglycemia and associated metabolic abnormalities on vascular structure and function [1]. Moreover, in recent years, it became apparent that vascular regeneration is impaired in diabetes, at least in part through pauperization of bone-marrow-derived progenitors [2]. Indeed, evidences accumulated to support the existence of circulating progenitors for several phenotypes not limited to the hematopoietic lineages and potentially important for the cardiovascular system [3]. Thus, endothelial [4], smooth muscle [5], osteoblast [6] and, possibly, cardiomyocyte progenitor cells [7] from the bloodstream have been described. These cells may have various protective or detrimental effects on vascular structure and function, although their quantitative contribution to cardiovascular biology is far from being definitely elucidated [3]. In parallel, the presence of mature circulating endothelial cells (CECs) is meant to represent an epiphenomenon of the ongoing vascular damage, as these cells are passively released from the vessel wall [8]. Importantly, most these cells have been implicated in the setting of diabetes and its chronic complication, suggesting a multifaceted contribution of blood-derived cells in the complex pathophysiology of diabetic micro- and macroangiopathy. These include reduced EPCs [9] and cardiomyocyte differentiation [10], increased generation of smooth muscle progenitor cells [11] and procalcific cells [6], paralleled by a high concentration of circulating shed CECs [12]. Very recent data suggest the existence of circulating pericyte progenitor cells (PPCs) [13]. Pericytes provide a variety of functions, such as capillary blood flow regulation, clearance and phagocytosis of cellular debris, and regulation of vascular permeability. Importantly, pericytes stabilize and monitor the maturation of endothelial cells by direct communication between the cell membrane and paracrine signaling [14]. They are recruited through the PDGF-B and PDGFR-Beta signaling, while PDGFR-Beta deficient mice display extensive vascular leakage, hemorrhage, and edema due to a defect of capillary coverage by pericytes [15, 16]. Interestingly, PPCs were found to be increased in patients and mice with malignant tumors and also increased after chemotherapy [13].

We hypothesized that PPCs play a role in the setting of diabetic microangiopathy. Based on this background and on the proposed role for PPC in response to cancer therapy, in this study we explored whether glucose control affects levels of circulating PPCs in type 2 diabetic patients, in relation to microvascular complications.

## 2. Materials and Methods

### 2.1. Patients

The study was approved by the Ethic committee of the University Hospital of Padova (protocol no. 1584P) and is registered in http://clinicaltrials.gov/ (NCT00699686). It was conducted in accordance with the Declaration of Helsinki and all patients provided written informed consent. Briefly, this was a trial of optimization of glucose control in type 2 diabetic patients poorly controlled on oral agents, with addition of basal insulin on top of their ongoing antihyperglycemic regimen. Insulin glargine and insulin detemir were compared in a randomized cross-over fashion during a 3+3 month period. The study design and clinical characteristics of the study population have been previously described [17]. The primary aim was to detect differences in the change of endothelial progenitor cells (EPCs) and circulating progenitor cells (CECs) levels in the bloodstream between the 2 insulin regimens. Out of a total of 42 patients, 21 were randomized to receive insulin glargine for 3 months and then insulin detemir for 3 months without washout, and 21 patients were randomized to the detemir-glargine treatment sequence. As a result of the study, we found that optimization of glucose control per se reduced CECs and other markers of endothelial damage, and increased EPCs, as markers of endothelial regeneration [17]. There was no difference in the effects of glargine versus detemir in terms of markers of endothelial damage and regeneration. This allowed us to consider the 2 insulin regimens and a single type of treatment. In parallel to EPCs and CECs, we also quantified circulating pericyte progenitor cells (PPCs) to evaluate the effects of glucose control on this cell type. PPC analysis was carried out in 38 patients and was unsuccessful in 4, due to technical reasons. Inclusion criteria were T2D with HbA1c >7.0% on oral agents, age 40–80 and presence of macroangiopathy (either coronary, peripheral or cerebrovascular artery disease). Exclusion criteria were T1D, acute hyperglycaemia, use of glitazones, DPP-4 inhibitors, cancer, any acute disease or infection, recent (within 3 months) surgery or cardiovascular intervention, serum creatinine >2.0 mg/dL, advanced liver disease, inability to provide informed consent, and pregnancy/lactation. All patients were characterized with anthropometric measures, evaluation of concomitant risk factors, diabetic complications and medications, as described elsewhere. Briefly, retinopathy was defined by a digital funduscopic examination as any degree of retinopathy according to the Early Treatment Diabetic Retinopathy Study (ETDRS) Research Group classification [18]. Nephropathy was defined by measuring urinary albumin/creatinine ratio on 3 different samples and the estimated glomerular filtration rate according to the MDRD equation [19]. Neuropathy was defined according to classical symptoms and signs, eventually confirmed by electromyography. 

### 2.2. Flow Cytometry

**Figure 1:**
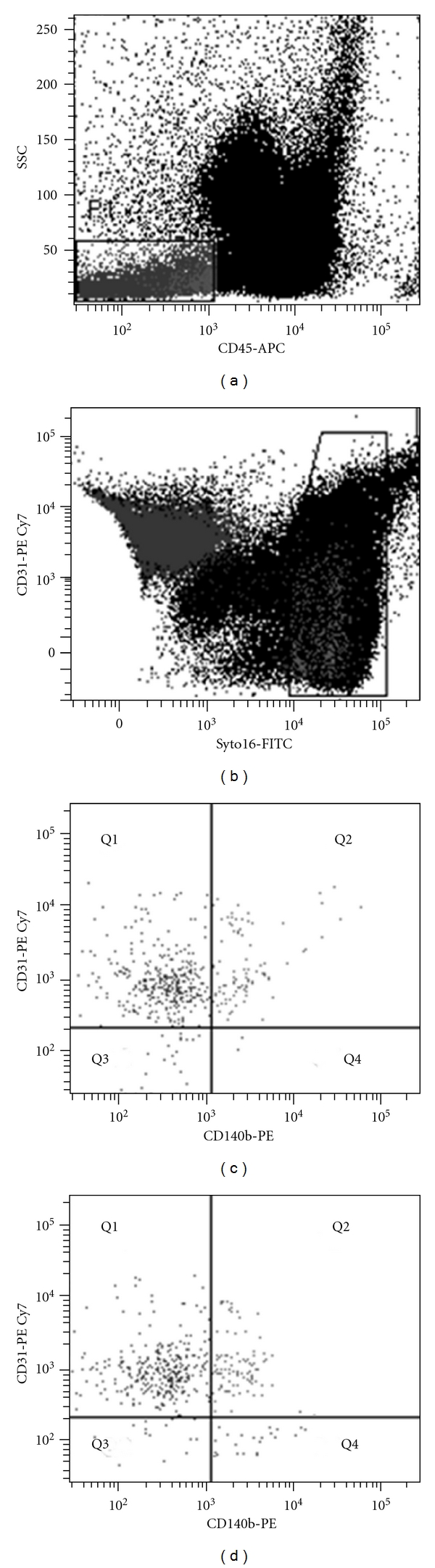
The gating strategy for enumeration of circulating PPCs. (a) Peripheral blood mononuclear cells were first gated into the CD45-negative fraction to exclude hematopoietic cells. (b) The total Syto16+ population of nucleated cells was selected to avoid inclusion of contaminating red cells, platelets, and debris in the analysis. (c, d) The resulting population was analyzed for expression of CD31 and CD140b. Panel (c) shows a case with low baseline PPCs (Syto16+CD45−CD31−CD140b+ cells), while (d) shows the same case 3 months after initiation of glucose control.

 Analysis was performed on frozen peripheral blood mononuclear cells according to a standardized protocol. PPCs were evaluated by six-color flow cytometry following an approach recently validated in our laboratory for the enumeration of CECs with some modifications [13]. PPCs were defined as Syto16+CD45−CD31−CD140b+ events. The nuclear staining Syto16 was used to discriminate between nucleated cells, platelets, and cell debris. The panel of monoclonal antibodies used included anti-CD45 (to exclude hematopoietic cells), anti-CD31 (an EC differentiation marker), and anti-CD140b (PDGFR-Beta). All antibodies were from Becton Dickinson (BD, Mountain View, CA). Cell suspensions were evaluated after cell recovery by a FACSCanto (BD). After acquisition of at least 1 × 10^6^ cells per blood sample, analyses were considered as informative when adequate numbers of cells (i.e., >100) were collected in the PPC enumeration gates. PPCs were defined as nucleated cells, negative for the hematopoietic marker CD45 and the EC marker CD31 and positive for CD140b. The gating strategy is illustrated in Figure 1. This definition identifies circulating cells not belonging to either leukocyte populations (CD45-neg) or shed endothelial cells (CD31-neg) and expressing the pericyte marker CD140b (PDGFR-Beta).

### 2.3. Statistical Analysis

 Data are expressed as mean ± standard error for continuous variables or as percentages for categorical variables. Comparisons between two groups were performed using two-tail Student's *t*-test for continuous variables or the chi-square test for categorical variables. To assess changes of PPC levels over time, we used the analysis of variance (ANOVA) for repeated measures with post-hoc paired *t*-tests. Statistical significance was accepted at *P* < 0.05.

## 3. Results

### 3.1. Patient Characteristics and Effects of Glucose Control

**Figure 2:**
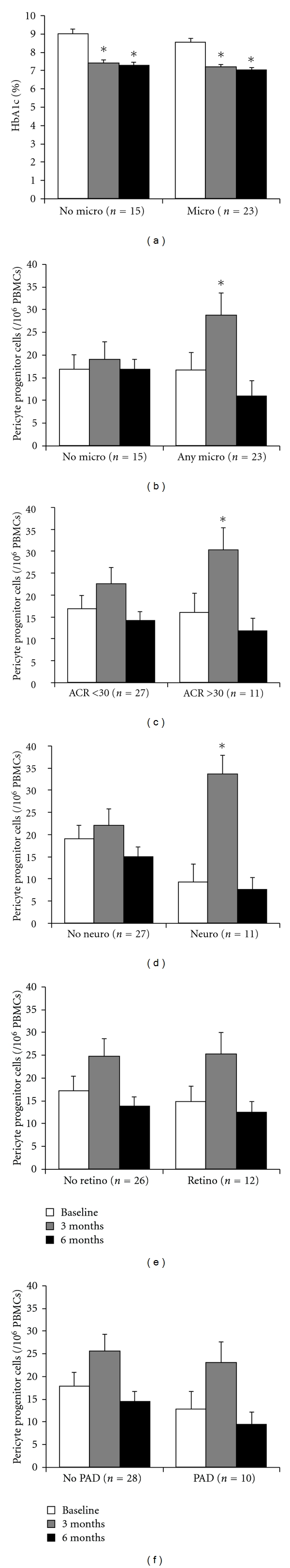
Effects of glucose control on HbA1c and PPCs. (a) There were no differences in HbA1c levels in patients with and without microangiopathy during time (*P < 0.05 versus baseline). (b) Increase in PPC levels was seen only in patients with microangiopathy (ANOVA *P* < 0.05; *post hoc *P* < 0.05). (c–f) Patients were divided according to the presence of micro-/macroalbuminuria, neuropathy, retinopathy, and peripheral arterial disease (PAD): a significant PPCs increase was detected in patients with urinary albumin-creatinine ratio (ACR) >30 mg/g and in the presence of neuropathy (ANOVA *P* < 0.05; *post hoc *P* < 0.05).

The characteristics of the 38 patients included in the study and divided by the presence/absence of microangiopathy are resumed in Table 1. Microangiopathy was defined as the presence of anyone among retinopathy, nephropathy (micro- or macroalbuminuria with or without renal failure), and neuropathy. Besides retinopathy, microalbuminuria, and neuropathy, differences between the two groups regarded lower HDL cholesterol levels and higher incidence of peripheral arterial disease (PAD) in patients with microangiopathy. These patients were subjected to optimization of glucose control for 6 months by means of addition of a basal insulin therapy according to a protocol described elsewhere. As there were no differences between glargine and detemir in the effects on endothelial markers of damage and regeneration, these treatment regimens were considered altogether as a single intervention. On average, HbA1c dropped from 8.8 ± 0.2% to 7.2 ± 0.1% (*P* < 0.001) indicating good optimization of glucose control, and 17 patients (45% of total) reached a HbA1c level of 7.0% of lower. Between the two groups (w/wo microangiopathy), there were no differences in baseline HbA1c levels or achieved HbA1c during the intensification protocol at 3 months (no microangiopathy 7.4 ± 0.2; microangiopathy 7.2 ± 0.1; *P* = 0.30) and 6 months (no microangiopathy 7.3 ± 0.2; microangiopathy 7.1 ± 0.1; *P* = 0.17; Figure 2(a)).

### 3.2. Changes in PPC Levels during Optimization of Glucose Control

 Circulating PPCs were measured at baseline, 3 months and 6 months. In the entire study population of 38 subjects, there was a trend toward increased PPC levels at 3 months versus baseline, which was not statistically significant (*P* = 0.29). There were no differences in PPC levels according to type of insulin used (*P* = 0.74 in the analysis for cross-over design). When patients were divided according to the presence or absence of microangiopathy, we found that PPC level remained unchanged during the entire course of the study in patients without microangiopathy, while it significantly increased at 3 months only in patients with microangiopathy (*P* = 0.01 using post-ANOVA *t*-test; Figure 2(b)). Among the 3 different types of microangiopathy that were considered, presence of micro-/macroalbuminuria (Figure 2(c)) and neuropathy (Figure 2(d)) were associated with PPC increase at 3 months, while retinopathy was not significantly discriminative of patients that increase PPC levels during the glucose control protocol (Figure 2(e)). Interestingly, in all cases, PPC levels returned to baseline at 6 months. As a control experiment, we also divided patients according to the presence/absence of PAD, which was more prevalent in patients with microangiopathy, and found that there was no differences in the trend of PPC levels over time in the two groups of patients (Figure 2(f)). The same was for coronary and cerebrovascular disease, which showed no correlation with PPC levels over time (not shown). Concentration of HDL cholesterol was not associated with change in PPC levels during the study (not shown).

## 4. Discussion

In this study, we found that in type 2 diabetic patients with microangiopathy glucose control is associated with a transient increase in circulating PPC levels.

Mounting evidence suggests that multilineage circulating progenitor cells have a variety of implications in diabetes and its complications. After endothelial progenitor cells (EPCs), smooth muscle progenitors, osteoblast precursors, and cardiomyocyte progenitors [4–6, 20], recent data now suggest the existence of circulating pericyte progenitor cells (PPCs) [13]. These cells have been identified and isolated from human or murine peripheral blood and reside in the nonhematopoietic (CD45-neg) compartment, and are distinct from CECs as they lack endothelial antigens (CD31-neg), but express the typical pericyte marker CD140b (PDGFR-Beta). This antigenic phenotype supports the pericytic origin, while electron microscopy confirmed their progenitor-like morphology, with high nucleus/cytoplasm ratio, rough endoplasmic reticulum cisterns and centrioles and absence of Weibel-Palade bodies typical of CECs [13].

In the setting of diabetic complications, pericytes may play an important role. Pericytes are an important component of the neurovascular unit both in the central and peripheral nervous system and may intervene in the pathogenesis of peripheral neuropathy, through the modulation of vasa nervorum [21]. Moreover, glomerular mesangial cells, which play a central role in the pathobiology of diabetic nephropathy [22], are specialized pericytes [23]. Finally, pericyte loss is one of the earliest features of diabetic retinopathy and the consequent defective endothelial coverage of retinal capillaries favors microaneurysmatic dilation and increased permeability [24]. Therefore, the study of PPCs may have important implications in the setting of diabetic microvascular complications.

Interestingly, PPCs were found to be increased in patients and mice with malignant tumors and also increased after chemotherapy [13]. Therefore, we analyzed whether the level of circulating PPCs is influenced by optimization of glycemic control in type 2 diabetic patients in relation to the presence of microangiopathy. In a cohort of 38 patients in which HbA1c was drastically reduced by insulin therapy, a significant increase in PPC level at 3 months was detected only in the presence of microangiopathy. Of note, this increase was transient, as cell counts returned to baseline at 6 months. Importantly, the PPC increase occurred during the first 3 month period, when HbA1c dropped markedly and then stabilized for the subsequent 3 months, suggesting that glucose control was the driver of PPC increase. We found that nephropathy and neuropathy were associated with PPC mobilization, while retinopathy was not. This is probably due to the fact that most patients had mild nonproliferative retinopathy and that a stratification for retinopathy severity was impossible, as groups of patients were too small. Moreover, the systemic levels of PPCs may not reflect processes ongoing within the central nervous system.

There are several potential implications of our present findings. First, it is possible that glucose control induces a mobilization of bone-marrow-derived PPCs, as previously shown for EPCs [25]. These cells would then function to stabilize blood vessels and counter the progression of diabetic microangiopathy. However, the study of GFP+ bone marrow chimeric mice suggests that murine PPCs are derived from peripheral tissues and not from the bone marrow [13]. Therefore, non-bone-marrow sources of these regenerative cells should be postulated [26]. The Madeddu's laboratory has clearly demonstrated that PPCs can be isolated from the saphenous vein and display potent cardiovascular regenerative activity [27, 28]. At present, we can only speculate on the mechanisms that induce PPC mobilization: it has been previously documented that circulating progenitor cells are recruited from the bloodstream to the perivascular space through the SDF-1/CXCR4 axis, whence they are mobilized by VEGF [29]. As insulin has been reported to stimulate VEGF and to interact with PDGFR (CD140b) signaling [30, 31], these growth factors may be important. A transient release of tissue PPCs induced by glucose control may also reflect regression of pathologic vascular structures in organs hit by diabetic microangiopathy, just as it happens in cancer chemotherapy. Regression of microvascular lesions owing to lower oxidative stress and inflammation achieved by glucose control [32] may also be responsible for passive mobilization of these cells from tissues to the bloodstream. Alternatively, the transient PPCs increase may be related to the worsening of diabetic microangiopathy that is sometimes induced by rapid glucose control [33, 34]. Unfortunately, owing to the relatively short duration of our study, it is impossible to determine whether the increase in PPC was associated with a favorable or unfavorable evolution of microangiopathy.

This study has other limitations, including the relatively small sample size and, importantly, the incomplete characterization of circulating PPCs. Indeed, it must be noted that cogent demonstrations that these Syto16+CD45−CD31−CD140b+ cells truly belong to the pericyte lineage and act as progenitors are still missing for the following reasons. First, there is no surface antigen that can unequivocally identify pericyte lineage cells. Second, we found a small degree of coexpression of the pericyte marker NG2 [35] by circulating CD140b cells (not shown), suggesting that the pericytic phenotype of PPCs is incomplete. Additionally, it is not clear how PPCs are related to CD34+CD140b+ cells, which Schober et al. identified as perivascular smooth muscle progenitors cells related to the severity of cardiac allograft vasculopathy [36]. Finally, selective culture of PPCs is needed to test their phenotype, proliferative potential, and function in vitro and in vivo.

## 5. Conclusions

Despite these drawbacks, the interpretation of our results lends to several intriguing speculations on the pathophysiology of diabetic microangiopathy and response to therapy. The mobilization of PPCs induced by amelioration of glucose control deserves a special attention in relation to the evolution of microangiopathy over time. Further studies are required to reach a better characterization of PPCs and to understand their relationships with diabetic complications.

## Figures and Tables

**Table 1:** Patients characteristics. *P* values are shown for paired Student's *t*-test or the chi-square test as appropriate. ACEi/ARB denotes angiotensin-converting enzyme inhibitors or angiotensin receptor blockers.

Characteristic	Without microangiopathy	With microangiopathy	*P*
Number	23	15	—
Age (years)	62.7 ± 2.4	67.2 ± 1.2	0.08
Sex male (%)	68.8	81.8	0.36
BMI (kg/m^2^)	29.5 ± 1.3	27.2 ± 0.6	0.10
Waist (cm)	103.5 ± 3.3	99.8 ± 2.2	0.35
Baseline HbA1c (%)	9.0 ± 0.3	8.6 ± 0.2	0.19
Concomitant risk factors			
Total cholesterol (mg/dL)	183.1 ± 6.5	176.8 ± 9.7	0.61
HDL cholesterol (mg/dL)	51.3 ± 2.9	43.2 ± 2.1	0.026
LDL cholesterol (mg/dL)	105.6 ± 5.2	97.1 ± 8.5	0.43
Triglycerides (mg/dL)	131.1 ± 14.5	181.8 ± 25.2	0.11
Smoking habit (%)	0.0	13.6	0.13
Hypertension (%)	93.8	81.8	0.29
Complications			
Retinopathy (%)	0.0	45.5	<0.01
Microalbuminuria (%)	0.0	50.0	<0.01
Neuropathy (%)	0.0	40.9	<0.01
Peripheral arterial disease (%)	6.3	36.4	0.03
Coronary artery disease (%)	18.8	27.3	0.55
Cerebrovascular disease (%)	75.0	77.2	0.82
Medications			
Metformin (%)	93.8	81.8	0.29
Sulphonylureas (%)	68.8	68.2	0.97
Aspirin (%)	68.8	86.3	0.19
Statin (%)	56.3	68.2	0.46
ACEi/ARBs (%)	87.5	63.6	0.10
Other antihypertensives (%)	68.8	63.6	0.75
